# Executive Functioning in Highly Talented Soccer Players

**DOI:** 10.1371/journal.pone.0091254

**Published:** 2014-03-14

**Authors:** Lot Verburgh, Erik J. A. Scherder, Paul A.M. van Lange, Jaap Oosterlaan

**Affiliations:** 1 Dept. of Clinical Neuropsychology, VU University Amsterdam, BT Amsterdam, The Netherlands; 2 Dept. of Social and Organizational Psychology, VU University Amsterdam, BT Amsterdam, The Netherlands; Universidad de Granada, Spain

## Abstract

Executive functions might be important for successful performance in sports, particularly in team sports requiring quick anticipation and adaptation to continuously changing situations in the field. The executive functions motor inhibition, attention and visuospatial working memory were examined in highly talented soccer players. Eighty-four highly talented youth soccer players (mean age 11.9), and forty-two age-matched amateur soccer players (mean age 11.8) in the age range 8 to 16 years performed a Stop Signal task (motor inhibition), the Attention Network Test (alerting, orienting, and executive attention) and a visuospatial working memory task. The highly talented soccer players followed the talent development program of the youth academy of a professional soccer club and played at the highest national soccer competition for their age. The amateur soccer players played at a regular soccer club in the same geographical region as the highly talented soccer players and play in a regular regional soccer competition. Group differences were tested using analyses of variance. The highly talented group showed superior motor inhibition as measured by stop signal reaction time (SSRT) on the Stop Signal task and a larger alerting effect on the Attention Network Test, indicating an enhanced ability to attain and maintain an alert state. No group differences were found for orienting and executive attention and visuospatial working memory. A logistic regression model with group (highly talented or amateur) as dependent variable and executive function measures that significantly distinguished between groups as predictors showed that these measures differentiated highly talented soccer players from amateur soccer players with 89% accuracy. Highly talented youth soccer players outperform youth amateur players on suppressing ongoing motor responses and on the ability to attain and maintain an alert state; both may be essential for success in soccer.

## Introduction

How can one optimally identify young talented athletes? This is a key challenge in sports, because identifying them at a young age helps them to promote their further development by selectively offering them the best training facilities, coaching, and support [1]. In general, a sport talent is defined as a young athlete who performs better than peers during training and competition at a young age, and who has the potential to reach the adult elite level [2]. Identification of talented athletes is also important in view of the high Olympic aspirations of many countries and to boost sports participation in the society [3]–[5]. For team sports, talent identification is complex because many skills (e.g. tactics, athletic skills and social interaction) may influence overall team performance, and skills of athletes may complement each other. The key question therefore is which skills are most important to the identification of talent.

In the past decades, most research on talent identification in team sports focused on physiological, anthropometrical, and technical characteristics of talented players. For example, it has been shown that physiological factors such as sprint performance, anaerobic capacity and interval endurance capacity discriminate between youth elite and non-elite field hockey and soccer players [6]–[8]. However, physiological and anthropometrical factors (for example body height, body mass and percentage body fat) appeared to be not predictive for future successful performance in soccer (see for a review [9]).

Recent evidence supports the importance of cognitive functions for sports performance. A meta-analysis by Mann et al. [10] showed that experts in various sports such as soccer and field-hockey, perform better than non-experts on sport-specific perceptual-cognitive tasks, such as visual fixation duration and quiet eye period. Also on basic attention and perception tasks, athletes perform better than non-athletes (see for a meta-analysis [11]). However, little is known about higher order cognitive functions in sports talents, especially in children and adolescents.

Higher order cognitive functioning, the so-called executive functions, might be cognitive functions that are particularly relevant for talent identification in sports. Executive functions manage other more basic cognitive functions (e.g. visuospatial perception) [12] and involve functions such as inhibition of behavior, attention, and working memory [13]. Executive functions might be important for successful performance in various sports, because these functions facilitate adaptation to new or changing situations, attention, as well as recall of game strategies [14]. A first attempt to measure executive functions in high division and lower division adult soccer players with a *non-sport specific task* was done by Vestberg and colleagues [15]. In that study, a Design Fluency task [16] was used, which is a multicomponent measure of executive functioning. Results showed that both groups scored better on Design Fluency compared to the normative mean, and that the high division players outperformed lower division players. Moreover, results showed a relationship between Design Fluency and the number of assists and goals a player scored two seasons later [15]. The authors suggested that high performance on executive functioning is important for soccer and may even predict future success. However, that study was limited by the use of one single measure of executive functioning that did not allow isolation of specific executive functions. Furthermore, only adult soccer players participated, whereas in view of talent identification and development it might also be interesting to examine executive functions at young ages.

The present study is among the first that investigates executive functioning in children who are playing soccer at the highest level. Three important components of executive functioning were examined in highly talented young soccer players: motor inhibition (the ability to suppress an ongoing motor action), various aspects of attentional functioning and visuospatial working memory (the ability to maintain and use relevant visuospatial information over time). We hypothesize that in sports such as soccer, motor inhibition plays an important role because motor actions frequently must be suppressed due to rapidly-occurring changes in the field (e.g. ball and player positions) [17]. Furthermore, excellent attentional focus is required and gathering relevant information in space plays an important role in order to cooperate with teammates, anticipate on the behavior of teammates, opponents and the ball [18]. Efficiency of three different networks of attention will be measured in the present study: alerting (to attain and maintain an alert state), orienting (to direct attention towards sensory input) and executive attention (to resolve conflicts between responses) [19], [20]. In addition, visuospatial working memory may be important for choosing positions and mentalizing possible game options. Two subsystems of working memory will be examined: The visuospatial sketchpad that holds visuospatial information over time and the central executive, the most complex component of working memory that enables attentional focus and coordinates tasks [21]. We compared highly talented soccer players of a youth soccer academy to amateur youth soccer players and it was hypothesized that highly talented soccer players would outperform amateur soccer players on all executive function measures.

## Methods

### Ethics Statement

The study was approved by the local ethical committee of the Institutional Review Board of the VU University Amsterdam. All participants and parents and/or legal guardians were informed about the procedures of the study before giving their written informed consent prior to participation.

### Participants

Eighty-four male highly talented soccer players (mean age 11.9, SD 2.2) and 42 age-matched amateur soccer players (mean age 11.8, SD 2.3) participated in the study. Every two highly talented soccer players were age-matched with one amateur soccer player with a maximum age difference of four months. Both groups ranged in age from 8 to 12 years. At the time of the study, all talented soccer players followed the talent development program of the youth academy of a Dutch Premier League club. Players at this youth academy have previously been selected by scouts on basis of several qualities (subjectively rated using broad criteria) such as technique (e.g. kicking the ball), athletic skills (e.g. speed) and tactics (e.g. positioning). Amateur soccer players were recruited from an amateur soccer club in the same geographical region as the Premier League club. The Dutch youth soccer system has nine levels and the players from the highly talented player group all play in the first level, which involves competition at the highest possible level for their age. The amateur soccer players that participated in the study were recruited from diverse teams and played on average in the 6^th^ level (range 4 to 9). The highly talented soccer players thus played on average 5 levels higher as compared to the amateur soccer players (range 3 to 8). Furthermore, at the time and in the region of our study, 79,118 boys between 8 and 12 years of age played soccer. Only approximately 300 boys between 8 and 12 years of age (from the 79,118 boys playing soccer) played were selected for the talent development program within one of the professional soccer clubs in this region that competitive season. Those boys were evaluated by scouts at their amateur club and when being considered very talented they were invited to enroll in the talent development program of the professional youth academy. Thus, the players from the highly talented group were among the best 0.004% of all players in the geographical region. Participants were free of known behavioral, learning and medical conditions that might impact performance on the executive function measures and were excluded when they had an IQ<70. Demographics of both groups are displayed in Table 1.

**Table 1:** Demographic results of highly talented soccer players and amateur soccer players.

	Highly Talented soccer Players	Amateur soccer players	Statistics
**Age, M (SD)**	11.9 (2.3)	11.8 (2.2)	*F* (1,125) = .05, *p* = .83
**Right handed, %**	88.1	91	Χ^2^(1, N = 126) = 1.61, *p* = .70
**Age started soccer, M (SD)**	5.2(1.4)	6.7 (1.5)	*F*(1,125) = 30,49, *p*<.001
**Physical activity h/week, M (SD)**	16 (6.4)	18.5 (6.9)	*F* (1,125) = 2.9, *p* = .09
**Estimated full-scale IQ (SD)**	93.7 (11.5)	95.6 (13.7)	*F* (1,125) = 5,5, *p* <.05

*Note:* Values shown are the mean and standard deviations on each measure. IQ = Intelligence Quotient.

### Materials

#### Executive functions measures

Adequate psychometric validity and reliability have been found for all executive function measures (e.g. [19], [22], [23], [24]).

#### Motor inhibition

To measure motor inhibition, the Stop Signal task [24] was used. The task involved go trials and stop trials. Go trials consisted of a drawing of an airplane presented in the center of the computer screen either pointing to the right or to the left and requiring a spatially compatible response on one of two response devices. A fixation point preceded the go stimulus. Stop trials consisted of a go trial and a stop signal (a white cross superimposed on the airplane), presented after to the airplane. Participants were instructed not to press either of the two buttons, when they saw the stop signal. The stop signal was presented after presentation of the airplane with an initial delay of 175 ms. If the response to the go stimulus was inhibited successfully, the delay was lengthened by 50 ms. If the subject failed to inhibit the response, the delay was shortened by 50 ms. This resulted in an average success rate around 50% on stop trials (for a detailed description of the paradigm see [25]). The task consisted of two practice blocks and three experimental blocks. The first practice block consisted of 32 only go trials. The second practice block consisted of 32 trials including 25% stop trials. Experimental blocks consisted of 64 trials and also included 25% stop trials. The dependent variable that reflects the latency of the inhibitory process is stop signal reaction time (SSRT). SSRT was calculated by subtracting average stop signal delay time from mean reaction time (MRT) calculated for correct responses on go trials. Shorter SSRTs reflect a faster and more efficient inhibitory process. Additional dependent variables derived from the Stop Signal task were MRT and the percentage of errors. Additional dependent variables derived from the Stop Signal task were MRT (measuring response speed) and the percentage of errors (measuring response accuracy). Errors were commission errors (left-right mistakes) on go trials, or omission errors on go trials (failing to press either button).

#### Visuospatial Working Memory

The visuospatial sketchpad and central executive were measured using an adapted version of the task developed by Bergman-Nutley et al. [26]. The forward condition was used to assess the visuospatial sketchpad. Here, participants were asked to reproduce a sequence of yellow circles that was presented in a 4×4 grid on a computer screen. Difficulty level was increased during the course of the task by increasing the span and by manipulating the position of the stimuli after every two trials. The difficulty level with two circles gave 2 points and passing at least one item on the more difficult sub level of the difficulty level with two circles gave 2.5 points and so on [27]. Two trials were administrated for each difficulty level, and the task was terminated when the participant failed to accomplish both trials on a sub level.

The central executive was assessed using the backwards condition where participants had to reproduce the sequences of yellow circles in the backward order. For both components of working memory, the total number of correct responses multiplied by highest sub level passed was included in the analyses, with the total score of the forward condition as a measurement of the visuospatial sketchpad and the total score of the backwards condition as a measurement of the central executive [24].

#### Attention

A modified child-friendly version of the Attention Network Test [19] was used to assess alerting and orienting attention ([28] for a detailed description). A fixation point was shown in the center of a computer screen. Then, participants were instructed to respond as quickly and accurately as possible by pressing either the left or the right response button corresponding to the side of the screen where a soccer goal appeared (the target stimulus). Neutral trials contained no cue at all and the target was presented instantaneously. Alerting trials contained a neutral cue that was presented in the center of the screen following the fixation point. Orienting trials contained a cue that was a referee pointing to the position where the soccer goal would appear. The experiment consisted of 168 trials, divided in four blocks, with one practice block of 24 trials and three experimental blocks of 48 trials each.

To assess the executive network, participants completed a modified version of the Flanker task [29]. The target stimulus was a black arrow against a white background, presented in the center of a computer screen, pointing either to the left or right. This target was flanked on either side by two black arrows pointing in the same direction (congruent trials), in the opposite direction (incongruent trials), or by black horizontal lines (neutral trials). Participants were required to respond as quickly as possible to the target by pressing on the left or right button corresponding to the direction the target was pointing to. The task consisted of one practice block with 12 trials and two experimental blocks of 36 trials.

Efficiency of the alerting network was calculated by subtracting MRT obtained on correct alerting trials from MRT on correct no-cue trials. Gain in MRT on alerting trials as compared with neutral trials was used as a measure of alerting attention. Efficiency of the orienting network was calculated by subtracting MRT on correct spatial trials from MRT on correct alerting trials. The gain in MRT on orienting trials compared with alerting trials was used as a measure of the ability to direct attention (orienting attention). Efficiency of the executive network was calculated by subtracting MRT on correct congruent trials from MRT on correct incongruent trials from the Flanker task. This difference in RT was used as a measure of the ability to actively ignore irrelevant information (executive attention).

#### Full-scale IQ estimation

Full-scale IQ was estimated by the Wechsler Intelligence Scale for Children III [30]. Two subtests (Vocabulary and Block Design) were administered, which correlate within the.90 range with full-scale IQ [31].

#### Physical activity estimation

Involvement in physical exercise might be beneficial for executive functioning, as has been shown by the meta-analysis of Verburgh et al. [32] which includes four studies including the pioneering work of Hillman and colleagues.

Therefore, physical activity (e.g. cycling to school, playing outside) during a typical week was assessed using a physical activity questionnaire [33]. This questionnaire consists of 13 questions (e.g., ‘How many days a week are you going to school walking or cycling?’) and measures the amount of physical activity. Participants were required to indicate how many days per week and how many minutes per day they participated in each of the activities listed. Adequate reliability and validity have been reported for the questionnaire [33].

### Procedure

Executive function measures were administered in the same order for each participant. There were single sessions for each individual participant, administered by trained assessors using standardized instructions. The total time for administration of the tests (including full-scale IQ estimation and the questionnaire) was about 1.5 hour. Data of the highly talented soccer players were collected at the training centre of the soccer academy. Amateur soccer players were tested in a quiet room at the soccer club. Data were collected during the competitive soccer season.

### Statistical analyses

SPSS version 20.0 [34] was used for all statistical analyses. Five outliers (0.5%) with z≥3.29 were transformed to a value one unit smaller than the most extreme non-outlier using a Van der Waerden transformation [35]. Technical difficulties or not speaking fluent Dutch resulted in missing data for some tasks. Missing data for all variables was less than 5% (n = 2 data points of executive attention, n = 1 value of alerting attention, n = 1 value of orienting attention and n = 6 of IQ) and were replaced by expectation maximalization [35]. Dependent measures derived from the executive function measures were converted into z scores. Possible group differences in physical activity, age started with soccer and IQ were tested using univariate analyses of variance (ANOVA) and Pearson correlations within each group were performed to determine the possible relationship between those variables and executive function measurements. Dependent variables derived from the executive function measures were subjected to univariate ANOVA's with group as between subjects factor (two levels: highly talented soccer players, amateur soccer players). Alpha was set at.05 and effect sizes were calculated in terms Cohen's *d* with values 0.20, 0.50 and 0.70 referring to small, moderate and large effects respectively [36]. Finally, stepwise logistic regression was used to predict group membership from those executive function measures significantly distinguishing between highly talented soccer players and amateur soccer players. Covariates were entered in the model when there were significant group differences for a variable and when it was correlated with possible predictors. The logistic regression consisted of a forward stepwise procedure in which the possible predictors were entered together. The algorithm terminates when all of the predictors have been entered into the equation or when addition of any of the remaining predictors does not significantly enhance classification to one of the groups.

## Results

**Figure 1:**
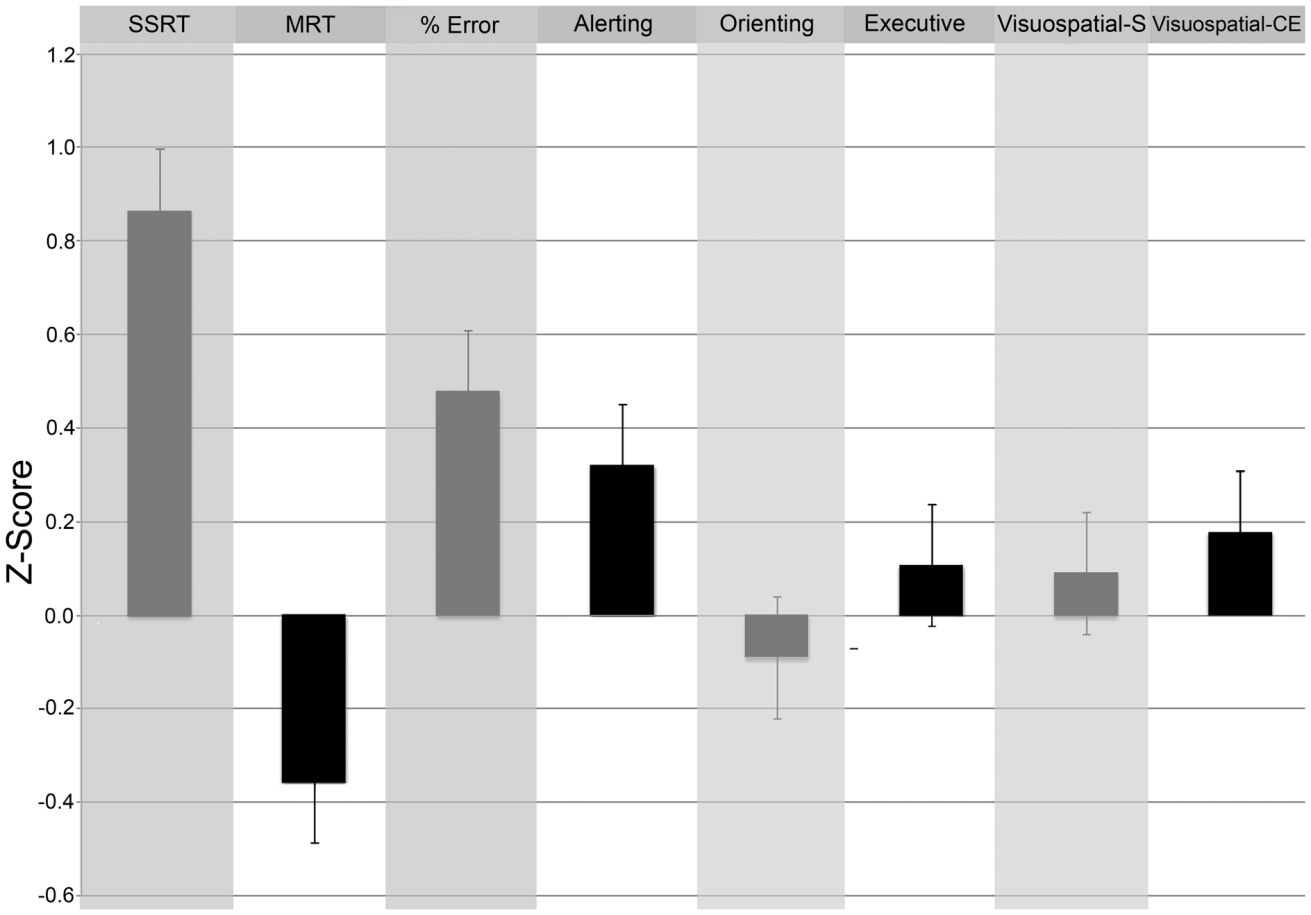
Executive function performance of highly talented soccer players as compared to amateur soccer players. Higher z scores are indicating better performance as compared to a mean z-score of zero in the amateur soccer player group. SSRT  =  stop signal reaction time; MRT  =  mean reaction time on go trials of the Stop Signal task; % errors  =  percentage of errors of the Stop Signal task; Alerting =  Alerting attention; Orienting  =  orienting attention; Executive  =  executive attention; Visuospatial-S =  visuospatial sketchpad; visuospatial-CE =  visuospatial central executive.

Demographic variables of both groups are presented in Table 1. The results for the executive function measures are presented in Figure 1.

Results revealed that amateur soccer players spent more time on physical activities than highly talented soccer players, but this difference was not significant (*F*(1,124) = 2.97, *p* = .09, *d* = .03). There were significant group differences for age at onset of playing soccer and IQ (*F*(1, 124) = 30.49, *p*<.001, *d* = .20, and *F*(1, 124) = 4.38, *p*<.05, *d* = .04 respectively), where the highly talented soccer players started earlier with soccer and showed a lower mean IQ. None of the executive functions measures was meaningfully related with starting age (*r*s<.21, *p*s>.06 for the highly talented soccer players, and *rs*<.17, *ps*>.31 for the amateur soccer players). Therefore, age at onset of playing soccer was not included as a covariate in subsequent analyses. The dependent variables for visuospatial sketchpad and visuospatial central executive correlated significantly with IQ (*rs*>.27, *ps*<.01). None of the other dependent measures correlated meaningfully with IQ (*rs*<.17, *ps*>.11). There is a debate on whether IQ should or should not be covaried, because there is (partial) overlap between executive functioning and IQ [37], [38] and IQ may be a possible inherent group characteristic [39]. Although results remained essentially unchanged with or without adjustments for IQ, results for visuospatial sketchpad and visuospatial central executive are covaried for IQ, which are described below.

### Motor inhibition

Univariate analyses of variance (ANOVA) showed group differences on stop signal reaction time (SSRT) and percentage of errors, indicating that the highly talented soccer players showed superior motor inhibition and committed less errors than the amateur soccer players (*F*(1, 124) = 25.08, *p*<.001, *d* = .89, and *F*(1, 124) = 7.84, *p*<.01, *d* = .52), respectively). Interestingly, highly talented soccer players showed slightly slower mean reaction times (MRTs) on go trials as compared to amateur soccer players (*F*(1,124) = 3.81, *p* = .05, *d* = .39). There was no evidence for speed-accuracy tradeoff. The Pearson correlation between percentage of errors and MRT was *r* = .13 (*p* = .24) in the highly talented soccer player group and *r* = .−14 (*p* = .38) for the amateur soccer players.

### Attention

The gain in MRT with presentation of the alerting cue as compared to no cue was larger in highly talented soccer players than in amateur soccer players (*F*(1,124) = 4.77, *p*<.05, *d = .42*), indicating greater use of the alerting cue. No group difference was found for orienting attention (*F*(1,124) = .54, *p* = .47, *d* = .19), indicating that the highly talented soccer players made similar use of the orienting cue as compared to the amateur soccer players. In addition, no group difference was found for executive attention (*F*(1,124) = 1.30, *p* = .26, *d* = .23), which indicates that both groups had similar increases in reaction times as a result of interfering information.

### Visuospatial (Working) Memory

No differences between groups were found for the visuospatial sketchpad (*F*(1,124) = .95, *p* = .10, *d* = .28), and central executive functioning (*F*(1, 124) = .24, *p* = .62, *d* = .09), indicating no differences in visuospatial working memory between highly talented and amateurs soccer players.

### Logistic Regression Analysis

The first step of logistic regression with SSRT was significant (*B* = −1.1, Wald *χ* ^2^ = 17.5, p<.001), with the model explaining 23% of variance (Nagelkerke *R* ^2^ = .23). In the second step, percentage of errors on go trials of the Stop Signal task entered the equation (*B* = −.52, Wald *χ* ^2^ = 4.7, *p*<.05); this model with SSRT and percentage of errors explained 28% of variance (Nagelkerke *R* ^2^ = .28). In the third step, alerting attention was included (*B* = 0.71, Wald *χ* ^2^ = 5.5, *p*<.05) and this model explained 33% of variance (Nagelkerke *R* ^2^ = .33). In the last step, MRT of the Stop Signal task was included (*B* = 0.44, Wald *χ* ^2^ = 3.6, *p*<.05). The final model explained 37% of variance (Nagelkerke *R* ^2^ = .37) and group membership of 78% of participants was correctly predicted, assigning 89% of subjects correctly to the group of highly talented soccer players and 55% of subjects to the group of amateur players.

## Discussion

The present study revealed several important findings on executive functions in young talented soccer players. More specifically, the findings indicate that motor inhibition, percentage of errors and MRT on the Stop Signal task, and alerting attention can accurately differentiate highly talented soccer players from amateur soccer players (89% correctly classified). Results of the Stop Signal task showed that highly talented soccer players outperform amateur soccer players in inhibiting an ongoing motor response. Our study is the first to demonstrate enhanced motor inhibition in highly talented soccer players at young age (mean age 11.9 years). Comparable results were found on motor inhibition skills in adult elite athletes [40]–[44]. Interestingly, the present results revealed that the highly talented soccer players showed slightly slower MRTs in the Stop Signal task than the amateur soccer players. On the other hand, the percentage of errors was smaller in the highly talented soccer player group. Hence, we suggest that the highly talented soccer players used a more conservative response strategy on the task. Speed-accuracy tradeoff (SATO) analyses within both groups revealed no relationship between MRT and accuracy, giving some support for our interpretation. In a recent study investigating cognitive functioning in professional adult volleyball players comparable findings were obtained [44], thereby providing initial evidence for the generalizability across two types of team-sport and across different levels of age or experience. Importantly, it has been shown that using a different strategy in the performance of a Stop Signal task does not influence the SSRT [45], because in this task speed of inhibitory control (SSRT) is independent of speed of response execution (MRT) [24].

It may be speculated that motor inhibition facilitates other (physical) skills which are required for performance on the field, such as agility. Agility is defined as “a rapid whole-body movement with change of velocity or direction in response to a stimulus” and the ability to change direction while sprinting is important for performance in open sports such as soccer, rugby, tennis and field hockey [46].

The analyses on the attentional networks revealed a group difference in alerting attention, indicating that the highly talented soccer players profit to a greater extent from temporal information to attain an alerting state (that allows quick responses), than amateur soccer players. Interestingly, highly talented and amateur soccer players profited to a similar extent from trials comprising both temporal and spatial information in a cue which appeared before target presentation. Information processing speed, as measured by MRT on trials without cues, did not differ between groups, which was shown before in other studies measuring simple reaction time in high and low skilled athletes [47], [48]. A recent study by Wang et al. [49] investigated temporal preparation in athletes before a warning signal and showed that tennis players outperformed sedentary controls on the ability to prepare for upcoming targets. Hence, it is possible that (talented) athletes have an enhanced ability to attain and maintain an alert state which allows fast reactions required in open sports.

In contrast to our hypothesis, no group difference was found for efficiency of the executive network. The executive network is known to measure the ability to suppress interfering information. Although this result might be counterintuitive, it has been found in studies investigating attentional skills in adult elite athletes [44], [11]. A possible explanation for this finding is that elite athletes are able to pay attention to a wider visual angle (i.e. higher peripheral awareness) in order to gather relevant perceptual information in the field in contrast to the narrower visual angle receiving attention in novices or amateur athletes [50]–[52]. As research has shown there are moderate correlations between measures derived from the Stop Signal task and the Flanker task [53], [54]. However, the Flanker task measures the control of perceptual interference and refers to a more initial stage of processing as compared to the Stop Signal task, which measures the ability to stop a prepotent response [55]. In the present paper, highly talented soccer players were not found to show superior inhibition of interfering information, but did show enhanced inhibiting of an already planned motor response as compared to amateur soccer players.

Results of the visuospatial working memory tasks indicated no group differences in visuospatial sketchpad and central executive functioning of visuospatial working memory, which contrasts with our hypothesis. Our finding receives support from a study by Furley and Memmert [56] using a spatial memory task in adult basketball players and showing no difference in visuospatial capacity between basketball players and non-athletes.

We should also acknowledge some limitations of the present research. The present findings do not allow any conclusions about the predictive ability of excellent executive functioning for future performance in soccer, because it is not yet known how many players from the highly talented group will become a professional soccer player. Therefore, there are several recommendations for future research. First, longitudinal measurements will be necessary to investigate the age-related development of executive functions in highly talented soccer players. In view of talent development it is important to know how executive functions of talented soccer players develop. Research has shown that for information processing speed, adult levels are reached around the age of 12, whereas inhibition and working memory continue to develop into the age of 15 [57]. With longitudinal measurements it is also possible to study causality between executive functioning and success in soccer. Furthermore, it would be informative to examine links between measures of executive function and measures of objective performance in soccer. In the study of Vestberg et al. [15] this was done by examining the number of goals and assists during the soccer competition. However, these performance measurements are difficult to measure in young players because positions of players vary and making goals is not the most important during a match. In future research, other objective outcomes of performance may be taken into account, such as the total number of correct passes of an individual player. Because it is not known whether superior executive functioning results in better soccer performance or if more time spent playing soccer leads to better executive functioning, future research should compare talented soccer players with young talented athletes of closed-skill sports (e.g. swimming) and matched peers who are not involved in any sports activities at all. Last, social functioning (e.g. social mindfulness, ego-orientation and leadership skills) of soccer players should be investigated because team results also depend in important ways on social interactions within the team [58].

An important implication of the present findings is that knowledge of executive function profiles of soccer players may be of value for talent identification in open sports. Furthermore, it may be suggested that knowledge of executive functioning of an individual soccer player may contribute to the developmental training plan of an individual player in professional soccer. Excellent performance in the top soccer level depends on factors such as the best possible physical fitness, equipment, and motivational processes. We emphasize the key role of neurocognitive functions as well, and suggest that even the slightest improvement in executive functioning might be important for soccer performance. Recently, a few studies have demonstrated the effectiveness of training executive functions in young adults [59] although generalization of training effects has not convincingly been shown and it should be investigated whether improvements in executive functions can be translated to soccer performance (e.g. [60], [61]).

In conclusion, highly talented youth soccer players outperform youth amateur players on suppressing an ongoing motor response and show superior ability to attain and maintain an alert state, both of which may be essential to success in soccer. Importantly, these results are also relevant to other team sports such as field volleyball, rugby, and basketball. Further research on executive functions, but also on other neurocognitive, motor and social functions, of talented team sport players and closed-skill athletes (e.g. swimmers) will enhance our understanding of what characterizes sports talents.
